# Hypervascular neck mass as the initial presentation of papillary thyroid cancer: A case report and review of current literature

**DOI:** 10.1016/j.ijscr.2019.12.010

**Published:** 2019-12-13

**Authors:** Slava Agafonoff, Shyam Allamaneni, Joseph Bernstein, Timothy Braverman, Imran Naqvi, Anastasya Chuchulo

**Affiliations:** a The Jewish Hospital, Department of Surgery, 4777 E. Galbraith, Cincinnati, OH, United States; b The Jewish Hospital, Department of Surgery, United States; c The Jewish Hospital, Department of Radiology, United States; d The Jewish Hospital, Department of Pathology, United States; e The Jewish Hospital, Department of Internal Medicine, United States; f Detroit Medical Center, United States

**Keywords:** Papillary thyroid carcinoma (PTC), Fine needle aspiration (FNA), Lymph nodes, Imaging, Upper respiratory infection (URI), Magnetic resonance imaging (MRI)

## Abstract

**Background:**

Papillary Thyroid Carcinoma presenting initially as a hypervascular mass is exceedingly rare. The objective of this paper was to present the rare pathology along with the utilization of multimodal imaging to establish a correct diagnosis along with current management and review of literature.

**Case presentation:**

We describe a 33 year old male who presented with a left-sided neck mass. CT with contrast of the neck revealed a hypervascular, ring enhancing, complex cystic lesion in the left sub-clavicular and supraclavicular neck deep to the SCM, with accompanying edema. The differential diagnosis was broad and difficult to achieve given the fine needle aspiration biopsy was inconclusive. Eventually, the patient underwent a total thyroidectomy with a left modified radical neck dissection, central neck dissection, and partial resection of sternocleidomastoid muscle.

**Conclusion:**

A large hypervascular mass presenting as a papillary thyroid carcinoma is difficult to discern but with combination of ultrasonography, soft tissue CT and angiography imaging, along with fine needle aspiration, and possible use of MRI would be successful in narrowing down the differential list to the final diagnosis of papillary thyroid carcinoma, supporting the need for punctual surgical intervention.

## Highlights

• Papillary thyroid cancer can present as an unusual hypervascular neck mass.
• Metastasis to regional lymph nodes is a common mechanism of thyroid cancer.
• FNA can be inconclusive in majority of case reports.
• CT, angio and MRI can be very helpful in narrowing the diagnosis of thyroid cancer.

## 1 Introduction

Thyroid cancer in America has been reported to have an incidence of 13.9 per 100,000 per year based on 2009–2013 cases, according to the NIH Surveillance, Epidemiology, and End Results (SEER) database, with strongest associated risk factors being prior history of radiation to the head or neck, as well as positive family history of thyroid malignancy. The most important prognostic factor in younger patients less than 50 years of age with thyroid tumors measuring more than 1 cm in size was found to be the presence of distant metastases. However, even with distal metastasis, for patients who had a disease-free period of over 3 years post treatment, the 10 year survival was calculated to be 96 %. Metastatic spread of papillary thyroid carcinoma (PTC) to cervical lymph nodes is common, as an initial presentation raising suspicion of numerous primary malignancies, providing clinicians with a diagnostic challenge. When non-metastatic causes for neck masses are added, the differential broadens significantly. We describe a case of PTC with an atypical clinical presentation of a hypervascular left sided neck mass with remarkable findings on CT angiography following upper respiratory infection-like symptoms [1,2].

This case report was reported in accordance with the SCARE criteria [13].

## 2 Case presentation

**Fig. 1:**
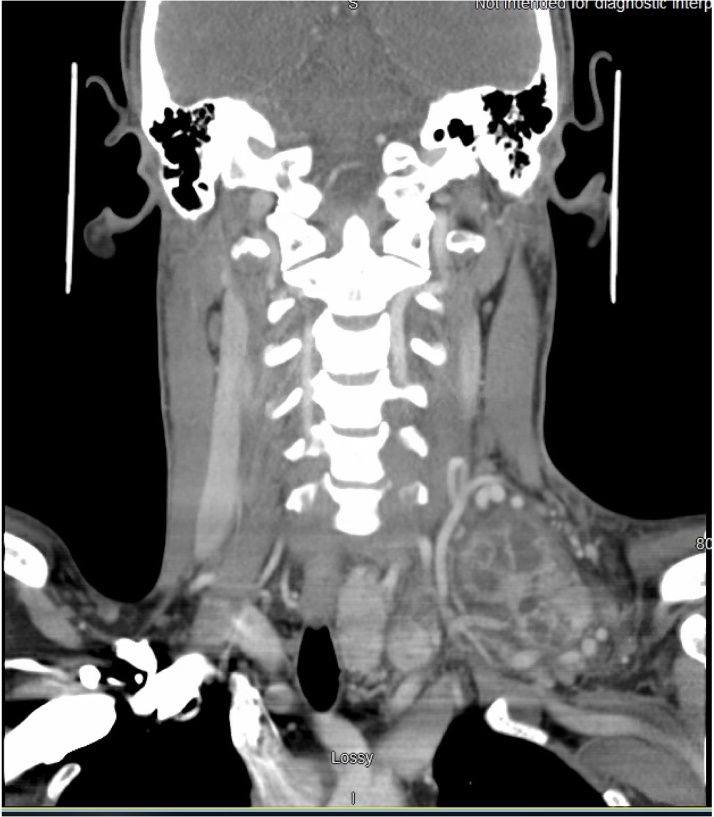
Hypervascular ring enhancing complex cystic lesion in the left subclavicular and supraclavicular neck deep to the sternocleidomastoid causing some swelling and edematous change. Tortuous enlarged vascular structures around the lesion are seen extending into the lesion.

**Fig. 2:**
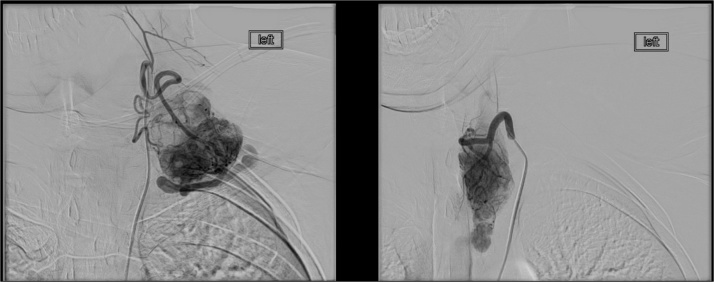
Hypervascular supraclavicular compartment mass with blood flow provided from the thyrocervical trunk was enlarged hypertrophic training vein into the left subclavian vein. Hypervascular nodule in the left lobe of the thyroid gland with blood flow from the thyrocervical trunk.

A 33 year-old Caucasian male with no significant past medical history presented to the Emergency Department with complaints of left-sided neck swelling after the onset of URI-like symptoms beginning four days prior to this visit. At that time, the patient experienced diffuse body aches, fevers, cough, and congestion that resolved in two days, with a subsequent self-discovery of the left neck mass. On further questioning, patient reported periodic night sweats and a ten pound weight loss over the past several weeks. He had no family history of endocrine malignancy and has never had any head or neck radiation exposure. On exam, patient was afebrile, the neck mass was mobile and mildly tender to palpation deep to the sternocleidomastoid muscle (SCM) and extended to the supraclavicular region, without erythema, palpable lymphadenopathy (LAD), or tracheal displacement. Laboratory evaluation showed a neutrophilic and monocytic leukocytosis, and thyroid function tests were normal. CT with contrast of the neck revealed a hypervascular, ring enhancing, complex cystic lesion in the left subclavicular and supraclavicular neck deep to the SCM, with accompanying edema (Fig. 1). Other findings were left paratracheal and prevascular lymphadenopathy, and an enlarged, heterogeneous thyroid gland, particularly of the left lobe. The initial differential included arteriovenous malformation (AVM), abscess, lymphoma, and non-hematolymphoid malignancy. It is well known that a variety of malignancies, especially of GI and thoracic origin, can metastasize to the left supraclavicular lymph nodes, therefore a CT of the chest, abdomen, and pelvis with contrast was requested, with no resulting evidence of primary malignancy. Ultrasound (US) imaging revealed a 3.5 cm heterogeneous left thyroid mass, with overall thyroid gland enlargement and high vascularity, the overall appearance compatible with Hashimoto’s thyroiditis. Initial fine-needle aspiration (FNA) biopsy of the neck mass was inconclusive owing to its high vascularity. Repeat FNA biopsy under US guidance of the left thyroid nodule was found to be variably cellular, containing variably-sized flattened clusters of cells with generous cytoplasm and variably sized nuclei, most with one or more chromocenters, and a few cells containing nuclear "grooves" and nuclear clearing/”vacuoles”, without an excess of “microfollicles”. The US-guided FNA biopsy of the left neck mass was significantly less cellular, but contained clusters of similar epithelium. The patient subsequently underwent angiography of the neck mass, which showed a prominent tumor blush and a large feeding vessel coming off the thyrocervical trunk, with an enlarged hypertrophic vein draining the blood supply into the left Subclavian vein (Fig. 2).

Following the above investigations, the patient underwent a total thyroidectomy with a left modified radical neck dissection, central neck dissection. The clavicular head of the sternocleidomastoid muscle was also resected as this was adherent to the mass. The internal jugular vein and carotid artery had to be mobilized to achieve identification of the entire tumor, with preservation of the spinal accessory nerve. The inferior thyroid pedicle and parathyroid glands were saved. Metastatic lymph nodes were dissected from levels I, III, IV, V, VI (central neck dissection) as were several large, firm nodes found within the carotid sheath.

The supraclavicular specimen grossly measured 6.6 × 5.7 × 3.4 cm and, when cut, revealed a tan to focally necrotic and hemorrhagic mass. Final pathology staging pT4aN1bM0.

Post-operatively the patient followed up with oncology and received radioactive iodine ablation therapy (131I), he was also started on levothyroxine 125 mcg daily and a low-iodine diet. Follow up thyroid scan revealed a single midline focus and received another dose of I131. At 1 year follow up, the patient had no surgical complications and his thyroglobulin level was < 0.1 with no signs of disease recurrence on follow up thyroid scans.

**Fig. 3:**
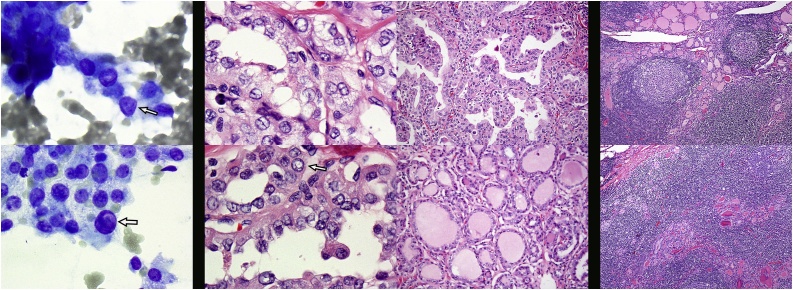
Right hand pair of images of Wright-Giemsa FNA with nuclear groove above (arrow) and intranuclear cytoplasmic inclusion below (arrow). Middle quartet of images, H&E tissue sections, the right hand pair with several nuclear grooves above and intranuclear cytoplasmic inclusion below (arrow), the left hand pair with papillary pattern above and follicular pattern below. Left hand pair of H&E tissue section images with Hashimoto’s pattern chronic lymphocytic thyroiditis in non-neoplastic thyroid gland.

Microscopic analysis of the thyroid revealed papillary thyroid carcinoma, intermixed classic and follicular variants in a background with prominent Hashimoto’s-pattern chronic lymphocytic thyroiditis accompanied by the expected diffuse and follicular lymphoid inflammatory pattern. The hypervascular mass was found to have fibroinflammatory tissue with foci of papillary thyroid carcinoma and surrounding dense mixed inflammation, necrosis and reactive features, and was positive for pan-keratin/CAM 5.2 stain, as expected for an epithelial tumor. Six lymph nodes were positive for metastasis (Fig. 3).

## 3 Discussion

Neck masses are quite common and present a plethora of primary/secondary and metastatic processes. The neck comprises one of the most concentrated areas of organs and structures in the body, any of which may produce a broad range of reactive, benign neoplastic, or malignant neoplastic processes, including inflammatory/reactive. The thyroid gland alone, the subject of this case, produces textbooks worth of information regarding benign neoplastic, reactive/inflammatory, and malignant masses, sometimes intermixed, as in our case, prior neck irradiation a risk factor for the last. Prognosis is excellent if the patient is younger than 40 with survival rates approaching 98 % at 5 years. For patients over 40 years old, survival decreases to 86 %. Additionally, primary tumors greater than 2 cm have worse prognosis [3,4].

After the presentation and initial evaluation of our patient, we were led to papillary thyroid carcinoma in the reasonably unusual presentation of a metastatic hypervascular mass. Minimal literature is available on this specific type of pathology.

Metastasis to regional lymph nodes is a common mechanism of thyroid cancer dissemination and occurs in 30–80 % of patients with PTC. In up to 40 % of all patients, the initial presentation can be enlarged cervical lymph nodes. However, only 2–8 % have distant metastasis, primarily to the lungs, and localized invasion of the larynx, trachea, and esophagus may occur. When making decisions regarding neck dissection, it is important that clinicians adequately evaluate cervical lymph node metastasis. Additionally, for patients who have an indication for therapeutic central or lateral compartment neck dissection with clinically apparent cervical lymph node metastases as detected by palpation, pre-operative imaging, especially in the diagnosis of PTC is vital [[3], [4], [5],^10^].

In a majority of case reports, FNA is inconclusive and diagnosis of PTC by final pathology after a more substantial biopsy (e.g. core) or excision has been the trend. In contrast to the case reports, FNA has been shown to have sensitivity of 95 % and negative predictive value of 96 % in the diagnosis of cancer. There are three main age groups to consider and their likely presentation. In the first, for those who are less than 15 years of age we consider congenital or inflammatory diagnosis. For those who are less than 40 years of age we must consider inflammatory conditions. Lastly, for those greater than 40 years of age we must suspect malignancy [7,9].

Cohen et al. demonstrated seven cases, all in which five had a positive FNA along with presentation of cervical cystic LN metastasis as the primary and singular manifestation of occult PTC; they used CT and ultrasound to assist in the pre-operative imaging. This brings up the next important point in which there are a variety of imaging modalities and radiographic clues that aid diagnosis and narrowing of the differential diagnosis, particularly in the setting of inconclusive FNA of the thyroid. Given that PTC is the most common thyroid malignancy in children and adults, it can present in variety of ways. A majority of calcified lymph nodes are secondary to thyroid cancer (40–50 %). Histologically, microcalcifications secondary to “psammoma bodies” (laminated classic calcifications) develop on the periphery of the lymph nodes, leading to peripheral calcifications on imaging studies, and important clue to PTC. Also, papillary thyroid carcinoma appears to be one of the very few malignancies that can completely cavitate a lymph node, by imaging mimicking a benign cyst with smooth, uniform wall. Although other cancers may similarly mimic a benign cyst on CT or MR, based on the literature, the most common carcinoma to do so is papillary thyroid cancer [1,3,5,6].

Approximately 5 % of patients with metastatic cervical adenopathy present with no known primary clinically. Juan et al. presented a case of a patient with consecutive metastatic calcified lymph nodes resembling a chain of rings on their chest CT. Som et al. presented various CT and MRI presentations occurring in thirteen separate cases of PTC, demonstrating that on CT, metastatic nodes can have multiple discrete calcifications, appears as benign cysts or hyperplastic/hypervascular nodes, or have areas of high attenuation reflecting intranodal hemorrhage/high concentrations of thyroglobulin. In comparison for MRI, nodes can have low to intermediate T1 and high T2 signal intensities, or high T1/T2 signal reflecting high thyroglobulin content [1,3,10].

Greco et al. defined important criteria for PTC metastasis by US or CT, including: enhancement, heterogeneity, calcification, cystic or necrotic changes, and a round shape. Size criteria for suspicion of metastasis include 15 mm in the long axes of the jugulodigastric and submandibular nodes and diameters exceeding 10 mm in all other cervical nodes, helpful in narrowing down the differential diagnosis. Takashima et al. evaluated MRI scans from 50 patients with PTC and concluded that cystic nodes or nodes 13 mm or more in minimum transverse diameter are highly suspicious. Other imaging clues on CT include a cystic node with a thin, uniformly enhancing rim, a homogenously enhancing node, node with variably sized small discrete calcifications, or node with areas of high attenuation (that represent hemorrhage), separately noting that even a hyperplastic-appearing node may contain metastases. Given the aforementioned findings, MRI would have been helpful in narrowing the differential diagnosis in our case [8,11,12].

Hypervascularity of a tumor, as discovered in our patient, must be evaluated prior to surgical intervention, requiring angiography. As Li et al. demonstrated, medullary carcinoma can present with massive AVM’s, supporting the need for pre-operative serum calcitonin level (given their high sensitivity and specificity) and angiography [10].

Asperstrand et al. performed a retrospective study on twenty-four patients with a vascular neck mass. MRI imaging was superior to CT and angiography in delineating cavernous hemangioma from hypervascular tumors. Contrast-enhanced CT may more clearly differentiate an MRI between capillary and cavernous hemangiomas of the head and neck, but cannot differentiate between capillary hemangiomas and hypervascular tumors [2].

Pathologically, PTC in cervical lymph nodes can vary widely in its architecture, in some cases with the classical appearance of largely papillary structures on fibrovascular stalks, associated with psammoma bodies (the result of degeneration of the tips of the papillae) and a paucity of colloid. Alternatively, it can grow primarily as follicles (follicular variant) and associated with colloid (thyroglobulin) production [3].

Additionally, but extremely rare, Wang et al. proved that PTC may arise in ectopic thyroid tissue at any location along the pathway of the thyroglossal duct from the foramen cecum to mediastinum, and ectopic thyroid carcinoma very rarely may present as a midline neck mass [8].

## 4 Conclusion

In light of normal TSH levels, the investigational modality of choice for thyroid nodules is ultrasonography followed by FNA. However, due to a conflicting clinical presentation in our case, which was initially associated with fever, chills, night sweats, recent weight loss, and mild leukocytosis, the initial differential was quite broad, including abscess, lymphoma, metastatic neoplasm, Hashimoto’s thyroiditis and arteriovenous malformation. A combination of ultrasonography, soft tissue CT and angiography imaging, along with fine needle aspiration, was successful in narrowing down the differential list to the final diagnosis of papillary thyroid carcinoma, supporting the need for punctual surgical intervention. Additionally, the use of MRI would have been useful in supplementary with the pre-operative diagnosis.

## Sources of funding

No source of funding.

## Ethical approval

Ethical approval is exempted as this is a case report.

## Consent

Written/Verbal informed consent was obtained from the patient for publication of this case report and accompanying images. A copy of the written consent is available for review by the Editor-in-Chief of this journal on request.

## Author contribution

Dr. Agafonoff did the bulk of the paper.

Dr. Braverman contributed to the pathology section of the paper.

Dr. Bernstein contributed to the radiology section of the paper.

Dr. Allamaneni/Dr. Naqvi/Dr. Chuchulo contributed to the writing of the paper.

## Registration of research studies

Not applicable.

## Guarantor

Guarantor is Dr. Slava Agafonoff.

## Provenance and peer review

Not commissioned, externally peer-reviewed.

## Declaration of Competing Interest

There are no conflicts of interest.
